# Assessing Inhibitory Control in the Real World Is Virtually Possible: A Virtual Reality Demonstration

**DOI:** 10.3390/bs12110444

**Published:** 2022-11-12

**Authors:** Francisco Rocabado, Jon Andoni Duñabeitia

**Affiliations:** 1 Centro de Investigación Nebrija en Cognición (CINC), Facultad de Lenguas y Educación, Universidad Nebrija, 28248 Madrid, Spain; 2 AcqVA Aurora Center, Department of Languages and Culture, UiT The Arctic University of Norway, 9019 Tromsø, Norway

**Keywords:** inhibitory control, virtual reality, cognitive assessment, Simon task, flanker task

## Abstract

Executive functions are the key ingredient for behaviour regulation. Among them, inhibitory control is one of the main exponents of executive functions, and in the last decades, it has received a good amount of attention thanks to the development of chronometric tasks associated with paradigms that allow exploring human behaviour when the inhibitory component is needed. Among the different paradigms typically used, the Simon and flanker tasks are probably the most popular ones. These have been subjected to modifications in order to assess inhibitory control from different perspectives (e.g., in different samples or in combination with different research techniques). However, its use has been relegated to classical presentation modalities within laboratory settings. The accessibility of virtual reality (VR) technology has opened new research avenues to investigate inhibition control with a high ecological validity while retaining tightly controlled lab conditions and good measurement accuracy. We present two cutting-edge modifications of the standard Simon and flanker tasks that have been adapted to real-world settings using VR and human-like avatars as target stimuli. Our findings show that virtual reality is a credible tool for testing inhibitory control with a high degree of transferability and generalizability to the real world.

## 1. Introduction

Executive Functions (EF) play the main role in providing adequate responses in those contexts in which automatic or instinctive responses are not enough [1], whether we find ourselves in a familiar situation or not. For instance, most daily human interactions with the environment, such as walking in a crowded space, visiting a new city, or running errands, will always require a suitable response to achieve the goal. Those responses come from a group of top-down cognitive processes that allow us to go through our daily routine efficiently. In the long run, EF have been proven to have an important weight on cognitive and psychosocial development [2], and they have been pinned as one of the main predictors of students’ academic performance and career trajectory development [3,4,5], hence, their importance regarding our physical and mental health.

Although there is no strict criterion for classifying EF, there is general agreement supporting a hierarchical framework that splits these abilities into three different core EF: inhibition, working memory, and cognitive flexibility [6,7,8]. From these core EF, higher-order EF abilities such as reasoning, problem-solving, and planning are distilled [6]. In this sense, even though all EF play a significant role in our life successes, their acquirement and trajectories seem to be linked to the needs of the specific developmental stages [9]. For instance, it comes as no surprise that core EF fulfil their development early in our lifecycle before adolescence, whilst higher-order EF continue their development through adolescence, reaching ceiling capacity in early adulthood [10,11].

The nature of inhibitory control and its value as a foundational unit of EF has been the focus of debate, receiving a great deal of attention among the scientific community [7,12,13]. Regardless of the controversy, there is no doubt about its value in our daily lives. Frequently, the characteristics of the environment will provide situations in which salient (bottom-up) stimuli will involuntarily attract our attention [14,15]. However, EF are typically capable of top-down distributing our attentional resources in a goal-oriented way so that we can selectively focus our attention while ignoring these and other interfering stimuli [16]. This way, our behavioural skills, empowered by EF, create the possibility of change and choice, which help us to willingly regulate emotional, cognitive, and behavioural responses without being under the control of environmental requirements, even when the circumstances might predispose another strong automatic response [6,13,17]. Thus, it is important for goal-oriented achievements that come from making the right choices whilst avoiding temptations by regulating impulsive decisions and behaviours [18,19].

**Figure 1:**
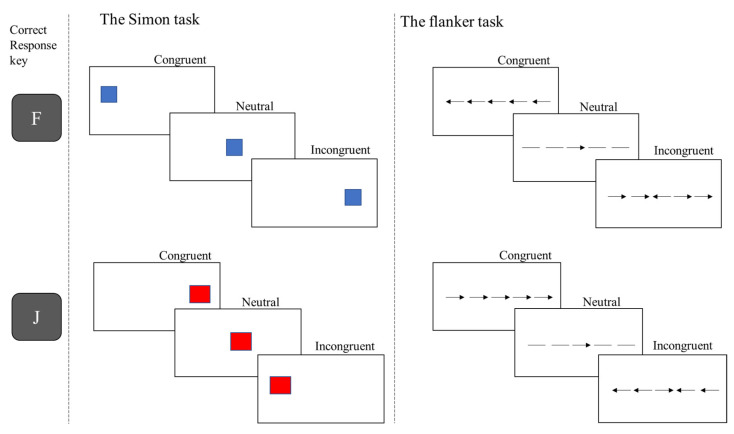
Typical trial conditions on the Simon and flanker task.

In the last 50 years, cognitive psychologists have been vested in elaborating controlled laboratory tasks to measure inhibitory control performance. So far, nonverbal interference tasks have attracted most of the research attention. Among these tasks, the Simon and flanker tasks [18] are the outstanding protagonists. In 1967, J. Richard Simon and Alan P. Rudell [20] introduced the first version of the task now known as the Simon task, where participants were given two simple instructions: press a given key (e.g., F) for stimulus type A and another key (e.g., J) for stimulus type B (see Figure 1). One stimulus is displayed at a time, and although its position is irrelevant to the task, participants’ responses are faster and more accurate when both target stimulus and response locations match than when they do not. This phenomenon, called the Simon effect of response facilitation, evidences a facilitating natural tendency to respond toward the stimuli’s location intermixed with the difficulty of inhibiting distracting and incongruous spatial information [20]. 

A few years later, in 1973, Barbara A. Eriksen and Charles W. Eriksen [21] created the well-recognised flanker task to complete the scarce literature in the field of visual search. As in the Simon task, the instructions to complete the task were simple: participants had to respond by pressing one of the two designated keys depending on each target stimulus (e.g., press F for stimulus A and J for stimulus type B; see Figure 1). However, unlike the Simon task, the target stimulus is presented in the centre of the screen and accompanied by flanking congruent (i.e., all presented stimuli are the same) or incongruent stimuli. In the original task, both the target and flanking stimuli were letters; every letter-string trial could display a target letter flanked by the same letter or by a different distracting letter. They found that reaction times were related to the level of competition flanking stimuli induced over the target stimulus. Thus, a target stimulus flanked by the same stimulus would produce more rapid and accurate responses than a target stimulus accompanied by a competitor, creating a response competition that we now understand as the flanker effect [22]. 

For many years these two tasks have helped capture individuals’ inhibitory control by collecting data from the two critical conditions’ trials (i.e., congruent and incongruent). Then, by contrasting the processing differences between reaction times and accuracy scores in the critical conditions, an interference score is measured; this is an indicative measure of a participant’s inhibitory control capacity. However, Simon and flanker task interference scores have shown low correlation coefficients between them [18]. Thus, they cannot be responsible for the same inhibitory control mechanisms involved during the task execution. However, there is general agreement that both tap into conflict monitoring and management. The distinction between them has been established on the basis of the type of conflict they generate and the hypothetical inhibition factor these conflicts represent—inhibition of prepotent responses in the case of the Simon task and resistance to distractor interference in the case of the flanker task [23].

Since their creation, both Simon and flanker tasks have evolved to adapt their applicability over different presentation modalities to reach a broad spectrum of samples [24,25,26,27,28]. As a result of these modifications, nowadays, inhibitory control data coming from these paradigms can be easily obtained with stimuli presented in the visual modality [29,30,31,32], the auditory modality [33,34], and the tactile modality [35]. Furthermore, some of these modifications have included materials adapted to different age groups [26,36] and special populations with and without medical conditions [37,38].

Aside from the differences in terms of taxonomy and the specific conflict monitoring demands in the two tasks, the debate about their capacity to measure inhibitory control in real-world activities remains open [18]. Not surprisingly, these tasks have been used almost exclusively in laboratory settings, and this could obviously yield laboratory- and task-specific effects [39]. In this sense, improved evaluation strategies are still to be developed to capture the underlying mechanisms of inhibitory control capacity in real-world activities with high precision and ecological validity. However, the difficulty of translating laboratory-studied paradigms into a more ecological environment without sacrificing control over extraneous variables is always a challenge for cognitive scientists [40].

The technological revolution experienced in the last decades made it possible to create Virtual Reality (VR) systems that are nowadays well implemented in different research fields, such as neuroscience and psychology, as reliable assessment tools [40,41]. New generation VR headsets are price-accessible and powerful enough to provide a high-end high-resolution stimuli presentation within realistic and interactive 3D environments, providing increased ecological validity, flexibility, sensory feedback, and performance recording [40,41,42,43]—see Gaggioli [44] for an early review of VR application. In fact, there are many successful examples of the use of VR as an assessment tool for skills related to EF, such as working memory and task-switching [45], cognitive load in navigation [46], memory [47], behavioural responses in eating disorders [48] and, more recently, cognitive function in terms of reasoning and attention switching [49].

Likewise, a few examples can be found regarding inhibitory control assessment in VR. Gupta and colleagues [50] translated the Simon task to a VR setting, aiming to assess alternative cues, response modalities, and VR ergonomics by projecting 2D stimuli through the VR headset. Furthermore, Olk et al. [51] successfully adapted the flanker task and increased its ecological validity by using daily objects instead of letters or arrows as stimuli. Roberts et al. [52] compared responses of the arrow version of the flanker task from a real laboratory setting and a matching replica scenario of the same laboratory in VR, showing that assessment from both scenarios is comparable and proves the utility of VR as an assessment tool. The most recent adaptation of the flanker task consists of projected typographic symbols over panels across a street [53]. As seen, both tasks have been successfully implemented in VR settings, essentially mimicking 2D scenarios in a 3D context.

To take full advantage of VR technology seeking to overcome current flanker and Simon tasks VR-adaptation limitations, in terms of task adaptation to real-life conditions (e.g., 3D objects, trial presentation), here, we propose a new strategy to assess inhibitory control by translating the classical tasks to a more realistic type of context, including humans as target stimuli and their behaviour as the critical conditions. We aim to explore this technology’s advantages in terms of natural task adaptability and as an assessment tool for inhibitory control in a close-to-reality environment without compromising assessment reliability by introducing a new user-friendly adaptation of both tasks within a complex real-world scenario that closely resembles a school classroom. Two experimental tasks were designed for each paradigm (Simon and flanker), one in 2D format and one being a 3D-adapted version of the same task.

## 2. Study 1: Simon Task

### 2.1. Participants

We recruited 36 participants from the Universitat de València with ages between 18 and 32 years old (M = 22.84, SD = 3.90; 14 females) and normal or corrected-to-normal vision. All participants were informed that data would be collected anonymously during the session and signed an informed consent form before starting. 

### 2.2. Materials and Procedure

#### 2.2.1. Simon Task 2D

All stimuli were presented on a 15.6” laptop screen from a distance of about 55 cm. The stimuli were one blue and one red coloured solid square displayed on a white background. Each trial began with a central fixation cross (+) for 500 ms. Participants were asked to press the “F” key if they saw a red square and the “J” key if they saw a blue square. They were asked to respond as fast as possible, avoiding making mistakes. A maximum response time of 3000 ms was accepted.

All trials pertained to three critical experimental conditions (congruent, incongruent, and neutral). Trials were defined as congruent when the target was displayed on the same side as the correct response key (e.g., a red square positioned on the left side of the screen). Incongruent trials were those on which the target location and the correct response were on opposite sides (e.g., a red square displayed on the right side of the screen). Finally, when the target was displayed centred on the fixation cross, these trials were defined as neutral (see Figure 1 for an illustration of the stimulus type employed in our task). 

The 2D experimental task was built using Gorilla Experiment Builder [54] and executed within the same web-based platform (www.gorilla.sc).

#### 2.2.2. Simon Task 3D 

**Figure 2:**
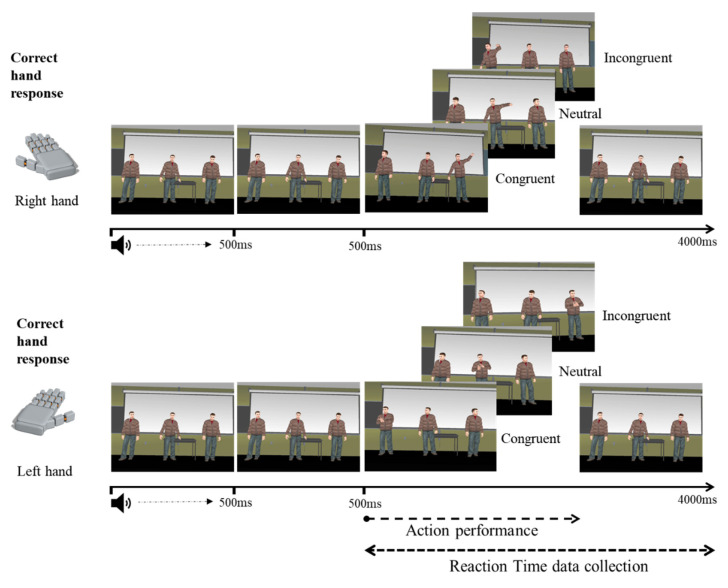
Time course of the 3D stimulus presentation in the VR adaptation of the Simon task.

The chosen stimuli consisted of three male human avatars that were programmed to perform two different actions: clapping hands and raising a hand. Each action was displayed within a virtual reality environment resembling a classroom scenario. One of the avatars was positioned in front of the participant’s point of view and centred within the scenario, and the other two were placed at his left and right sides within the field of view. All three avatars were displayed simultaneously and programmed to remain in an idle position. Each trial began with a neutral auditory stimulus for 500 ms that alerted participants of the beginning of a new trial. After 500 ms, one of the avatars would perform one of the programmed movements. As in the 2D version, a maximum response time of 3000 ms was permitted (see Figure 2). A video of the virtual environment and the task is provided at https://doi.org/10.6084/m9.figshare.19984631.

Within the virtual environment, each controller became a virtual hand; thus, participants held each virtual hand with the corresponding real hand and were asked to give quick and accurate responses by pulling the trigger in each controller to indicate if the avatar performing a movement was either clapping (left trigger) or raising a hand (right trigger). Similar to the 2D version of the task, all trials pertained to one out of three critical experimental conditions (congruent, incongruent, and neutral). Trials were defined as congruent when the moving avatars’ action was performed on the same side as the correct response trigger (e.g., leftmost avatar clapping). Similarly, incongruent trials were defined as those in which the critical avatar’s location and the correct response trigger were on opposite sides (e.g., the rightmost avatar clapping). Finally, when the moving avatar was centred on the participants’ point of view, these trials were defined as neutral.

The 3D experimental task was created using Vizard 6.0 [55], a Python-based software (Python v. 2.7.12; Python Software Foundation, https://www.python.org/). The experiment script was executed on a high-end gaming laptop (MSI GL76) computer equipped with an Intel Core i7-10750H (2.6 Hz), running Windows 10 operating system (64 bit), 32 GB RAM, and an NVIDIA GeForce RTX 2070 video card. To ensure and maintain high-performance connections between the PC and the VR HMDs, battery-saving settings were disabled. The 3D stimuli were presented through the HTC Vive Pro HMD [56] at 2880 × 1600-pixel resolution (1440 × 1600 per eye) and 90-Hz refresh rate, thus, providing 110 degrees of field of view and high immersive experience, made of a high-quality display and a stable tracking system [43].

Regardless of the task version (2D or 3D), all began with a practice period in order to familiarise participants with the task. This practice included 12 trials, 4 from each condition. After the practice, the experimental trials followed, including 48 trials per condition. Experimental trials were distributed across three blocks. Each block included 16 trials per condition that were randomly presented. Overall, the 2D task was completed in around 5 min and the 3D task in about 8 min. Between the two versions of the task, a 15-min distracting task was presented. The presentation of the two-task version was counterbalanced across participants.

### 2.3. Results

Collected data was wrangled in RStudio [57] and analysed with JASP [58]. Descriptive analyses were undertaken to ascertain reaction times and accuracy (see Table 1). Mean reaction times (RT) were computed for each condition and participant at a trial level by including only accurate responses. Additionally, participants’ RT that were below 100 ms and 2.5 SD faster or slower than the mean RT per condition or those associated with timed-out responses were rejected (2.94% of the data in the 2D modality and 1.86% of the data in the 3D version).

A sensitivity analysis was run to determine the predicted magnitude of the effect sizes needed to grant reliability to the observed findings [59,60,61]. G*Power 3.1 software [62] was used to run this analysis which was conducted using F tests with α = 0.05, power = 0.80, and a total sample size of 36 people as the input parameters. Given such a sample size, alpha, and estimated power, the sensitivity analysis revealed that we could detect values as low as f = 0.18 (F_critical_ = 2.27 (5, 170)); consequently, resulting F-values equal to or greater than the F_critical_ value were deemed significant and reliable. (Note that all the significant F-values reported in the current study at the *p* < 0.05 level were also significant when contrasted with the F_critical_ value.)

**Figure 3:**
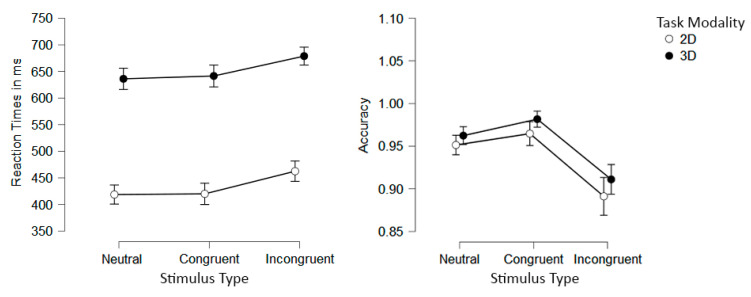
Mean RT (**left**) and accuracy (**right**) per Stimulus Type and Task Modality presentation conditions. Error bars represent 95% confidence intervals.

We carried out a 3 (Stimulus Type: congruent, incongruent, and neutral) × 2 (Task Modality: 2D and 3D) repeated-measures ANOVA on the RT data. A significant main effect of Stimulus Type was found (*F*[2, 70] = 62.016, *p <* 0.001, η_p_^2^ = 0.639). Post hoc analyses revealed that differences occurred between congruent and incongruent conditions (*M_Diff_* = −39.952, *SE* = 4.326, *p_bonf_* < 0.001), reflecting the classical Simon interference effect, and between incongruent and neutral conditions (*M_Diff_* = 43.296, *SE* = 4.326, *p_bonf_* < 0.001), showing an incongruency effect. No significant difference was found between congruent and neutral conditions (*M_Diff_* = 3.343, *SE* = 0.773, *p_bonf_* = 1). Additionally, the main effect of Task Modality was also significant (*F*[1, 35] = 189.223, *p* < 0.001, η_p_^2^ = 0.844), being latencies from the 3D modality larger than the 2D (*M_Diff_* = 218.470). Importantly, there was no interaction between Stimulus Type and Task Modality (*F*[2, 35] = 0.271, *p* = 0.277, η_p_^2^ = 0.008) (see Figure 3).

A similar repeated-measures ANOVA was performed on the accuracy scores from both tasks. When sphericity assumptions were violated, the Greenhouse–Geisser correction was applied. A significant main effect of Stimulus Type was found (*F*[1.652, 70] = 48.783, *p* < 0.001, η_p_^2^ = 0.582). Post hoc analyses showed significant differences between congruent and incongruent conditions (*M_Diff_* = 0.072, *SE* = 0.006, *p_bonf_* < 0.001; namely, a Simon effect) and between incongruent and neutral conditions (*M_Diff_* = −0.056, SE = 0.006, *p_bonf_* < 0.001; namely, an incongruence effect). No significant difference was found between congruent and neutral conditions (*M_Diff_* = 0.016, *SE* = 0.008, *p_bonf_* = 0.110). The main effect of Task Modality was also significant (*F*[1, 35] = 6.535, *p* = 0.015, η_p_^2^ = 0.157), being responses on the 3D modality more accurate than in the 2D modality (*M_Diff_* = 0.016). Finally, there was no interaction between Stimulus Type and Task Modality (*F*[1.458, 35] = 0.225, *p* = 0.728, η_p_^2^ = 0.006) (see Figure 3).

### 2.4. Discussion

The 2D and 3D versions of the Simon task showed a markedly similar response and accuracy pattern across all Stimulus Type conditions. In both settings, incongruent stimuli elicited longer response latencies compared to congruent and neutral stimuli, and classic Simon effects and incongruency effects were replicated both in the 2D and 3D versions of the paradigm. RT significantly differed between task versions, with participants showing shorter response latencies when the task was performed in the 2D context, an effect that can be easily explained by core differences in the stimuli presentation. Whereas in the 2D version of the task, the stimuli were displayed without a movement component, in the 3D version, participants had to hold their response until the avatar movement was evident. This interpretation aligns with the small difference between task versions found in terms of accuracy (which was not influenced by the time needed by the characters to initiate a movement) and with the similar pattern of effects found across versions occurring in all task conditions. In conclusion, our results showed that both the 2D and 3D tasks are equally capable of capturing participants’ inhibitory control towards prepotent responses as measured by the Simon interference effect.

## 3. Study 2: Flanker Task

### 3.1. Participants

For this experimental session, data from 46 participants from Nebrija University with ages between 18 and 52 years old (*M_age_* = 25.23, *SD* = 7.05; 31 females) were collected. All participants had normal or corrected-to-normal vision and no signs of cognitive dysfunction as measured by the Cognitive Assessment Battery (CAB)™ PRO (CogniFit Inc., San Francisco, CA, USA; https://www.cognifit.com/cab) and were naive to the purpose of the experiment. Before starting the experimental session, participants were informed that data would be collected anonymously, and they signed informed consent.

### 3.2. Materials and Procedure

#### 3.2.1. Flanker Task 2D

All stimuli were presented on a 15.6” laptop set at a distance of around 50 cm to the participants’ eyes. A series of arrays of five arrows were employed as experimental stimuli, including one central target arrow and two flanking arrows at each side. Each trial began with a central fixation cross (+) for 500 ms, followed by one stimuli array. The target stimulus was displayed for 3000 ms or until a response was given. If a response was given, this was followed by a between-trial blank space lasting for 500 ms. All participants were encouraged to keep their index fingers always positioned over the “F” key for the left hand and the “J” key for the right one on the keyboard, and they were prompted to respond as fast as possible while avoiding making mistakes. Participants’ task was to press the button corresponding to the direction of the central target arrow: they had to press “F” if the target stimulus pointed to the left and “J” when the target pointed to the right. Experimental trial conditions were defined as follows: congruent when all five arrows pointed towards the same direction, incongruent when the central and the flanking arrows pointed in the opposite direction, and neutral when dashes instead of arrows flanked the target arrow.

As in the case of the Simon task, the 2D version of the flanker task was built with Gorilla Experiment Builder [54].

#### 3.2.2. Flanker 3D Task

**Figure 4:**
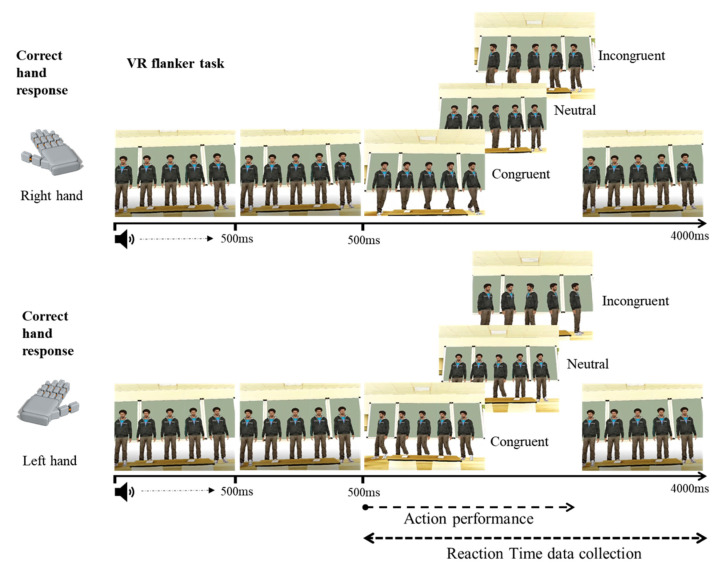
Time course of the 3D stimulus presentation in the VR adaptation of the Simon task.

Primary experimental stimuli consisted of five male human avatars programmed to spin to the left or the right. The programmed movement took place within 400 ms, and once the movement was completed, the avatar would return to its initial position. All five avatars were presented within a scenario that resembled a classroom. Following the same vein as in the classical flanker task, one of the avatars was positioned in the centre of the scene, in front of the participant’s point of view, and flanked by two avatars on each side. All avatars were displayed simultaneously in a synchronised idle position, separated by the same distance. However, to maximise the presence of an interference effect and based on pilot data from our lab, the target avatar was set to initiate its movement 200 ms after the flanker avatars did. This decision was taken after a series of pilot tests where all avatars spun at the same time. Results from this piloting included data from 35 participants and showed a complete absence of an interference effect in the reaction time analysis when all the avatars initiated the movement at the same time, most probably because of the saliency of the difference in movement of the central (target) avatar (*F*(2, 68)= 1.219, *p* = 0.302). An extract of the recording of this pilot study is provided as Supplementary Material at https://doi.org/10.6084/m9.figshare.19984631 for the readership to examine and experience the differences regarding the final setting. Each trial began with a neutral auditive stimulus that lasted 500 ms, alerting participants of the beginning of a new trial. To avoid stimuli overlapping, the experimental trial began 500 ms after the sound. Participants were asked to respond using the provided controllers by paying attention to the spinning direction of the central avatar: they were asked to pull the trigger of the left-hand controller if the target avatar spun to the left and to pull the right-hand controller if he spun to the right. Experimental trial conditions were defined as congruent when all five avatars spun in the same direction, incongruent when the central avatar and the flanker avatars spun in opposite directions, and neutral when the central avatar performed his movement while the flanker avatars maintained their idle position—note that responses were only collected once the central avatar spun. An illustrative schematic example is presented in Figure 4, and a video of the virtual environment and the task is provided at https://doi.org/10.6084/m9.figshare.19984631.

The 3D version of the flanker task was created using Vizard 6.0 [55], and stimuli were presented through the HTC Vive Pro HMD [56], mimicking the 3D data collection associated with the Simon task reported in Experiment 1.

In both 2D and 3D versions, experimental trials were distributed across three blocks, each including 16 trials per condition randomly presented. Both versions began with 12 practice trials (4 trials per condition). Overall, the 2D task lasted between 5 and 7 min, whereas the 3D task was completed between 8 and 11 min. A distractor task was placed between our two presentation modalities. The distraction task lasted 15 min. All experimental tasks were presented in a counterbalanced order between participants.

### 3.3. Results

All collected data were processed and cleaned in RStudio [57] and analysed in JASP [58]. Descriptive analyses were performed to ascertain RT and accuracy (see Table 2). Mean RT was computed for each condition and participant at a trial level, including only accurate responses. Moreover, participants’ RT that were equal to or below 100 ms and 2.5 SD faster or slower than the mean or those associated with timed-out responses were trimmed from any analysis (2.78 % and 2.48% of trimmed data from the 2D and the 3D task modalities, respectively).

Similar to the process followed in Study 1, in Study 2, we also performed a sensitivity analysis to determine the magnitude of the reliable effect sizes using G*Power 3.1 software [62] with α = 0.05, power = 0.80, and a total sample size of 46 individuals. The sensitivity analysis showed that we could detect values as low as *f* = 0.15 (*F_critical_* = 2.26 (5, 220)), and F-values equal to or greater than the F_critical_ value were considered significant.

**Figure 5:**
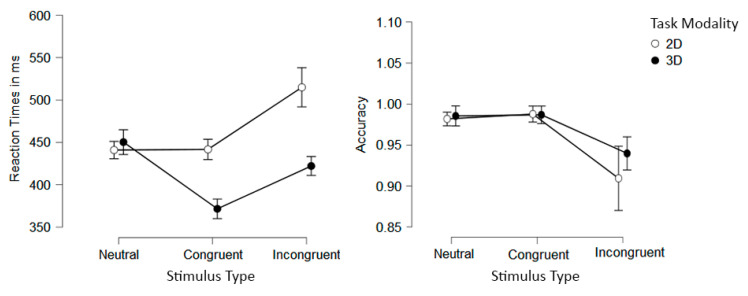
Mean RT (**left**) and accuracy (**right**) per Stimulus Type and Task Modality presentation conditions. Error bars represent 95% confidence intervals.

We first carried out a 3 (Stimulus Type: congruent, incongruent, and neutral) × 2 (Task Modality: 2D and 3D) repeated-measures ANOVA on the RT data. When sphericity assumptions were violated, the Greenhouse–Geisser correction was applied. A significant main effect of Stimulus Type was found (F[1.354, 60.923] = 67.938, *p* < 0.001, η_p_^2^ = 0.602). Post hoc analyses showed significant differences between congruent and incongruent conditions (namely, a flanker effect; *MDiff* = −61.910, *SE* = 5.370, *p*_bonf_ < 0.001), incongruent and neutral conditions (an incongruity effect; *MDiff* = 22.924, *SE* = 5.370, *p*_bonf_ < 0.001), and between congruent and neutral condition (a congruency effect; *MDiff* = −38.987, *SE* = 5.370, *p*_bonf_ < 0.001). Additionally, a significant main effect of Task Modality was found (F[1, 45] = 30.081, *p* < 0.001, η_p_^2^ = 0.401), with the latencies for the 2D modality being longer in comparison to the 3D (*MDiff* = 51.205). Finally, the interaction between Stimulus Type and Task Modality was also significant (F[1.317, 59.282] = 41.683, *p* < 0.001, η_p_^2^ = 0.481). Post hoc analyses showed that latencies were significantly larger in the 2D than in the 3D version for congruent and incongruent conditions (*p*_bonf_ < 0.001), but no statistical differences were found between Task Modalities for the neutral conditions (*p*_bonf_ = 1.00; see Figure 5).

For accuracy scores, a similar 3 × 2 repeated measures ANOVA was performed. A significant main effect of Stimulus Type was found (F[1.074, 48.341]= 28.740, *p* < 0.001, η_p_^2^ = 0.390). Post hoc analyses showed significant statistical differences between congruent and incongruent conditions (namely, a flanker effect; *MDiff* = 0.063, *SE* = 0.009, *p*_bonf_ < 0.001) and between incongruent and neutral conditions (an incongruity effect; *MDiff* = −0.059, *SE* = 0.009, *p*_bonf_ < 0.001); on the contrary, no significant difference was found between the congruent and neutral conditions (*MDiff* = 0.004, *SE* = 0.009, *p*_bonf_ = 0.697). No significant main effect on Task Modality was found (F(1, 45) = 1.524, *p* = 0.223, η_p_^2^ = 0.033). Finally, no interaction was found (F(1.079, 48.564) = 1.470, *p* = 0.235, η_p_^2^ = 0.032) (see Figure 5). 

A post hoc analysis was carried out to examine potential differences between the two types of movements involved in the VR experimental setting (i.e., hand raising or clapping), given that they had markedly different dynamics and execution times. Trials including clapping actions were responded to 141 ms faster than trials involving avatars raising their hands. While the magnitude of the differences across conditions were significantly reduced in the trials involving longer movements (namely, raising hands), the direction of the effects did not change and the interference effects remained, albeit to a lesser extent (55 vs. 13 ms). While this difference is of relevance in terms of the analysis of movement patterns, it does not alter the main conclusion of the results of this task, which stays as a valid real-life reinterpretation of the classic Simon task with the potential of reproducing incongruity effects with human-like characters and realistic movements.

### 3.4. Discussion

The classical flanker effect was replicated in the 2D task modality, in which incongruent trials generated longer latencies and higher error rates compared to both congruent and neutral conditions. Importantly, the 3D modality of the flanker task yielded strikingly similar results as well: incongruent trials generated longer latencies and larger error rates as compared to congruent trials, showing an instance of the well-known flanker effect with a dramatised VR version of the task.

Unlike the 3D Simon task used in Experiment 1, in Experiment 2, the sequence of programmed movements was not complex, which could explain the shorter latencies observed in the VR modality as compared to the 2D version, especially in the congruent and incongruent conditions. An effect that deserves mention is the relatively high RT associated with the items from the neutral condition in the 3D version of the task (450 ms vs. 422 ms in the incongruent and 372 ms in the congruent condition, respectively). This effect can be understood in the specific context of the 3D task we devised, in which participants first previewed the beginning of the movement of the flanking characters 200 ms before the target (central) character started to spin. In neutral trials, the flanking characters did not spin, and participants were most likely withholding responses, being conditioned by the expectancy of a trial beginning with the flanking avatars’ movement. Thus, it is reasonable to believe that participants would carefully wait during a neutral trial before giving any response. Hence, we interpret this condition-specific effect as a task-induced result that should not deviate the focus from the impact of the findings. Inhibitory control assessment by the flanker task has typically relied on the differential score between incongruent and congruent conditions, and here we showed that such conditions presented in a 3D realistic variant could perfectly capture participants’ resistance to distractor interference the same way the classical 2D version of the task does.

## 4. General Discussion

Virtual reality (VR) technology has become increasingly popular in recent years among healthcare providers, and its use for the assessment and training of cognitive skills has recently received a great deal of attention. In essence, VR allows users to interact with 3D environments using multiple senses, and in the current study, we demonstrate the usefulness of employing a head-mounted device (HMD) with a three-dimensional environment to generate a completely immersive experience in which specific aspects of inhibitory control can be correctly measured.

Regardless of the procedure [40,49,51] or the aim of the study [50,52,53], none of the previous VR adaptations of the Simon and flanker tasks were capable of making the most of the possibilities offered by virtual reality when transferring and testing these tasks in a creative, plausible, and realistic way. The current work represents a new ecologically valid alternative for assessing inhibitory control as a relevant construct of executive functions. We present two state-of-the-art variants of the well-known Simon and flanker tasks in a fully immersive quasi-realistic virtual environment that aims to translate classical laboratory paradigms to real-world situations.

Through two experimental studies, we have presented evidence showing that it is possible to capture a snapshot of certain cognitive processes as measured by reaction times and response accuracy by presenting stimuli in a context close to reality using VR. Stoffels and van der Molen’s [28] arrow representation of the flanker task used material that implied orienting directionality to generate the interference score between congruent and incongruent directions. In their seminal study, Bidet-Ildei and Bouquet [25] used a much more realistic scenario with human characters and relied on video sequences of running people to replicate this same interference effect. Based on this premise, our task aimed to generate the same conflict by relying on the orienting movement made by the avatars. This approach enables us to generate a more natural expression of the task that allows us to generate a more naturalistic assessment of inhibitory control that, as a result, helps us deepen a concept that, until now, has been limited to 2D scenarios. In this sense, our results show that both 2D and 3D presentation modalities are equally capable of capturing classic indices related to resistance to interference and inhibitory control. Our results open a path toward new possibilities for the real-world assessment of human inhibitory skills within real-life scenarios, situations, and activities that require interference control, such as following someone in crowded spaces or finding someone within a multitude. Interestingly, preceding studies have questioned the extent to which response inhibition takes place in a similar manner in VR and 2D settings, thus raising doubts about the degree of transferability of in-lab results to more naturalistic or realistic environments. For instance, Bailey et al. [63] found that children were less likely to inhibit responses within VR as compared to a classical 2D context and that VR settings elicited stronger social compliance behaviours towards the stimuli because of the saliency and realism of the visual materials. In a parallel line exploring the relevance of immersive scenarios and how they shape behaviour, Gomez et al. [64] found that graspable items have a stronger influence on attention and manual reactions as compared to static non-graspable representations of objects. These results suggest that the physical engagement that real objects provide in VR settings can modulate attention-related behaviours.

As we noted in the Introduction, there is an ongoing debate about the ability of tasks such as those used in the current study to quantify inhibitory control with high sensitivity and specificity, given the lack of convergence in the results obtained from different paradigms that are seemingly designed to measure the same construct [18]. One of the arguments that have been put forward to partially explain this absence of convergence between the results of executive function tasks and daily activities that are expected to also tap into the same inhibitory processes in real life is the artificial nature of laboratory tasks. We are confident that this study will open doors to future adaptations of classical cognitive tasks to more naturalistic scenarios. By doing so, we expect to contribute to the debate on the role of inhibitory control in daily life activities by showing examples of immersive real-life-like approaches to explore inhibition skills with tasks that elicit interference in a more naturalistic manner. Admittedly, our VR adaptations of the Simon and flanker tasks are just a first step in a long journey towards exploring the interaction between social or contextual factors and executive functions. Nonetheless, the demonstration that interference effects and inhibitory control can be effectively captured using human-like avatars allows us to move forward in our exploration of the variables of real-life scenarios that can modulate inhibitory skills, such as the properties of the human characters (e.g., gender, race, or age). In this line, it is worth noting that the flanker task adaptation by Bidet-Ildei and Bouquet [25] already suggested that the gender of the avatar influenced target recognition. Preceding studies have discussed the possible interaction between implicit biases and executive functions [65,66], but the unclear picture obtained so far may well be the result of the use of laboratory tasks with static representations of non-human elements in non-immersive contexts. Future VR adaptations of these and other related tasks could overcome these limitations regarding the naturality and plausibility of the stimuli and contexts. Additionally, future studies could explore the role played by some specific effects that otherwise could not be captured in classic 2D settings, such as effects derived from manipulations on the number of virtual avatars [67] or effects related to the distance between the participant and the avatars (e.g., effects related to the violation of the personal space [68,69]). We provide the first piece of evidence demonstrating that this is a feasible research avenue, and we acknowledge that much more work needs to be done to bring the designs even closer to daily-life activities.

Although there is still a long way to go in the process of standardising the use of VR systems as tools to deliver psychometric tests, the use of techniques that allow us to evaluate cognitive processes using chronometric-based behavioural responses with high levels of ecological validity will positively impact the quality of the data associated with the measurements. Since the 2020 announcement of Facebook’s intentions to move its platform into the metaverse, many R&D companies have started to allocate economic and technological resources to the advancement of the virtual universe, boosting the expansion of VR systems. In accordance with these changes, we firmly believe that future research should make an effort to create and validate adaptations of the well-established paradigms from cognitive science for VR platforms.

The use of VR for cognitive assessment incurs some costs that might slow down the generalisation and globalisation of this technology. We believe that the scientific community should move towards an open-source practice where tools and methods are shared. In this vein, we aim to provide the community with the use of these two new adaptations of the flanker and Simon task with the hope that it will positively impact our understanding of EF assessment. To this end, our scripts have been made available at https://doi.org/10.6084/m9.figshare.19984631. While there are still some rough edges in terms of the immersiveness of the VR scenarios that will certainly experience important changes in the following years, it is important to restate that these do not seem to significantly alter the results from the tasks: inhibitory skills and incongruity effects can be effectively measured in close-to-real VR scenarios.

## Figures and Tables

**Table 1:** Mean accuracy proportions and reaction time (RT) results per condition, task modality, and task. Standard deviation is presented in parentheses.

Task Modality	Stimulus Type	RT (in ms) *M* (*SD*)	Accuracy *M* (*SD*)
Simon 2D	Congruent	420 (75.53)	0.96 (0.04)
	Incongruent	462 (87.68)	0.89 (0.08)
	Neutral	419 (67.53)	0.95 (0.04)
Simon 3D	Incongruent	641 (110.06)	0.98 (0.02)
	Congruent	679 (100.75)	0.91 (0.06)
	Neutral	636 (102.64)	0.96 (0.03)

**Table 2:** Mean accuracy proportions and reaction time (RT) results per condition, task version, and task. Standard deviation is presented in parentheses.

Task Modality	Stimulus Type	RT (in ms) *M* (*SD*)	Accuracy *M* (*SD*)
Simon 2D	Congruent	441 (71.90)	0.98 (0.02)
	Incongruent	515 (123.58)	0.90 (0.14)
	Neutral	441 (69.60)	0.98 (0.02)
Simon 3D	Incongruent	372 (82.40)	0.98 (0.02)
	Congruent	422 (89.23)	0.94 (0.06)
	Neutral	450 (95.96)	0.98 (0.03)

## Data Availability

Data available at: https://doi.org/10.6084/m9.figshare.19984631.

## Supplementary Materials

The following supporting information can be downloaded at https://doi.org/10.6084/m9.figshare.19984631. Video samples of each task and VR .exe of each task.
